# *N*-Cyclo­hexyl-4-meth­oxy­benzene­sulfonamide

**DOI:** 10.1107/S1600536811009172

**Published:** 2011-03-15

**Authors:** Muneeb Hayat Khan, Islam Ullah Khan, Muhammad Nadeem Arshad, Shumaila Younas Mughal, Mehmet Akkurt

**Affiliations:** a Materials Chemistry Laboratory, Department of Chemistry, GC University, Lahore 54000, Pakistan; b Department of Physics, Faculty of Sciences, Erciyes University, 38039 Kayseri, Turkey

## Abstract

In the title mol­ecule, C_13_H_19_NO_3_S, the S atom has a distorted tetra­hedral geometry with an O—S—O bond angle of 120.39 (18)°. The cyclo­hexane ring has a chair conformation. In the crystal, mol­ecules are connected by inter­molecular N—H⋯O hydrogen bonds, forming zigzag hydrogen-bonded chains directed along the *c* axis.

## Related literature

For background to the biological activity of sulfonamides, see: Gennarti *et al.* (1994 ▶); Hanson *et al.* (1999 ▶); Moree *et al.* (1991 ▶); Ozbek *et al.* (2007 ▶); Rough *et al.* (1998 ▶); Siddiqui *et al.* (2007 ▶). For literature on sulfonamide derivatives, see: Akkurt *et al.* (2011 ▶); Aziz-ur-Rehman, Rafique *et al.* (2010 ▶); Aziz-ur-Rehman, Sajjad *et al.* (2010 ▶); Aziz-ur-Rehman, Siddiqa *et al.* (2010 ▶); Khan, Akkurt *et al.* (2010 ▶); Khan, Sharif *et al.* (2010 ▶). For puckering parameters, see: Cremer & Pople (1975 ▶).
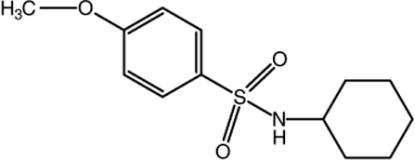


## Experimental

### 

#### Crystal data

C_13_H_19_NO_3_S
*M* *_r_* = 269.36
Orthorhombic, 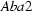

*a* = 17.2644 (12) Å
*b* = 20.4707 (16) Å
*c* = 7.9139 (5) Å
*V* = 2796.9 (3) Å^3^
*Z* = 8
Mo *K*α radiation
μ = 0.23 mm^−1^
*T* = 296 K
0.29 × 0.12 × 0.09 mm

#### Data collection

Bruker APEXII CCD diffractometer
10601 measured reflections
2704 independent reflections
1945 reflections with *I* > 2σ(*I*)
*R*_int_ = 0.047

#### Refinement

*R*[*F* ^2^ > 2σ(*F* ^2^)] = 0.048
*wR*(*F* ^2^) = 0.136
*S* = 1.02
2704 reflections
167 parameters
2 restraints
H atoms treated by a mixture of independent and constrained refinement
Δρ_max_ = 0.17 e Å^−3^
Δρ_min_ = −0.27 e Å^−3^
Absolute structure: Flack (1983 ▶), 829 Freidel pairs
Flack parameter: −0.05 (12)

### 

Data collection: *APEX2* (Bruker, 2007 ▶); cell refinement: *SAINT* (Bruker, 2007 ▶); data reduction: *SAINT*; program(s) used to solve structure: *SIR97* (Altomare *et al.*, 1999 ▶); program(s) used to refine structure: *SHELXL97* (Sheldrick, 2008 ▶); molecular graphics: *ORTEP-3 for Windows* (Farrugia, 1997 ▶); software used to prepare material for publication: *WinGX* (Farrugia, 1999 ▶) and *PLATON* (Spek, 2009 ▶).

## Supplementary Material

Crystal structure: contains datablocks global, I. DOI: 10.1107/S1600536811009172/xu5173sup1.cif

Structure factors: contains datablocks I. DOI: 10.1107/S1600536811009172/xu5173Isup2.hkl

Additional supplementary materials:  crystallographic information; 3D view; checkCIF report

## Figures and Tables

**Table 1:** Hydrogen-bond geometry (Å, °)

*D*—H⋯*A*	*D*—H	H⋯*A*	*D*⋯*A*	*D*—H⋯*A*
N1—H*N*1⋯O2^i^	0.85 (3)	2.09 (3)	2.913 (4)	161 (3)

Symmetry code: (i) 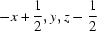.

**Fig. 1.**
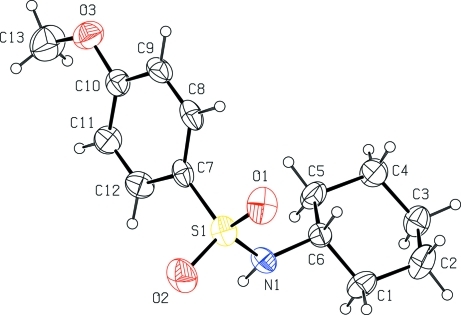
View of the title molecule, with atom numbering scheme. Displacement ellipsoids for non-H atoms are drawn at the 30% probability level.

**Fig. 2.**
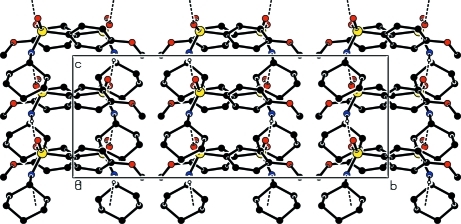
The packing and hydrogen bonding of (I) viewed down a axis. Hydrogen atoms that not involved in the hydrogen-bonding (dashed lines) have been omitted for clarity.

**Fig. 3.**
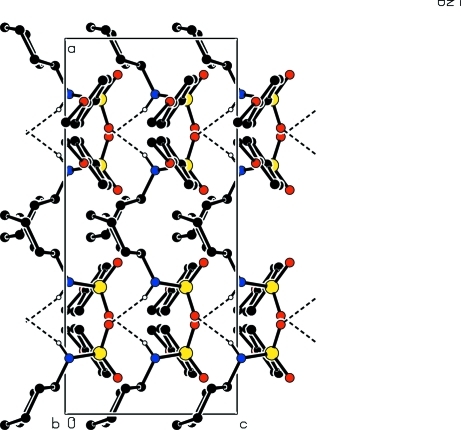
The packing and hydrogen bonding of (I) viewed down b axis. Hydrogen atoms that not involved in the hydrogen-bonding (dashed lines) have been omitted for clarity.

**Fig. 4.**
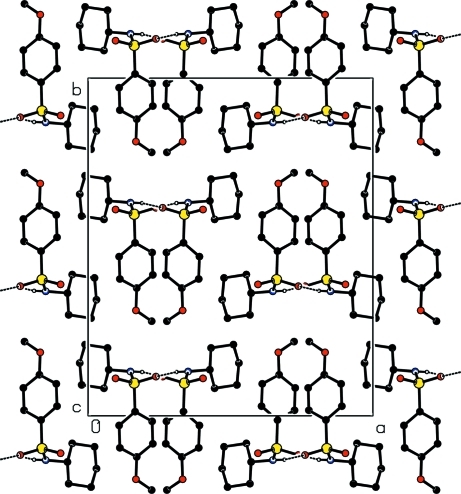
The packing and hydrogen bonding of (I) viewed down c axis. Hydrogen atoms that not involved in the hydrogen-bonding (dashed lines) have been omitted for clarity.

## supplementary crystallographic information

### Comment

Sulfonamides are familiar for their enormous potential as biologically active molecules (Hanson *et al.*, 1999; Moree *et al.*,1991; Rough *et al.*, 1998). They are being used as anti-microbial (Ozbek *et al.*, 2007), anti-convulsant (Siddiqui *et al.*, 2007), and for the treatment of inflammatory rheumatic and non-rheumatic processes including onsets and traumatologic lesions (Gennarti *et al.*, 1994). In continuation of our structural studies on various sulfonamide derivatives, herein we report the crystal structure of the title compound (I) (Akkurt *et al.*, 2011; Aziz-ur-Rehman, Rafique *et al.*, 2010; Aziz-ur-Rehman, Sajjad *et al.*, 2010; Aziz-ur-Rehman, Siddiqa *et al.*, 2010; Khan, Akkurt *et al.*, 2010; Khan, Sharif *et al.*, 2010).

As shown in Fig. 1, the S atom of the title molecule has a distorted tetrahedral coordination geometry, with S1—O1 = 1.424 (3), S1—O2 = 1.436 (3), S1— N1 = 1.586 (3), S1—C7 = 1.747 (4) Å, O1—S1—O2 = 120.39 (18), O1—S1—N1 = 108.09 (16), O1—S1—C7 = 107.08 (16), O2—S1—N1 = 105.37 (16), O2—S1—C7 = 106.18 (15) and N1— S1—C7 = 109.43 (16)°. The cyclohexane ring (C1–C6) has an almost ideal chair conformation and the ring-puckering parameters (Cremer & Pople, 1975) are Q_T_ = 0.559 (4) Å, θ = 1.8 (4) ° and φ = 338 (9)°.

In the crystal structure, adjacent molecules form zigzag hydrogen-bonded chains directed along the *c* axis, linking by intermolecular N—H···O hydrogen bonds (Table 1). The packing and hydrogen bonding of (I) are viewed down a, b and c axes, respectively, in Figs. 2, 3 and 4.

### Experimental

Cylcohexylamine (0.5 g, 6.494 mmol) was taken in 50 ml round bottom flask and added 10 ml of distilled water. After 5 minutes stirring at room temperature 4-methoxy benzene sulfonyl chloride was carefully added. The pH of the reaction mixture was maintained at 8 with 10% Na_2_CO_3_ solution. After 6 h of stirring at room temperature the TLC check confirmed the completion of the reaction. The reaction mixture pH was reduced to 3 with 3 M HCl, product precipitated out was filtered and dried. Dried precipitates were dissolved in methanol for crystallization (yield: 87%).

### Refinement

In the last cycles of the refinement, 3 reflections (2 0 0), (1 2 0) and (0 2 0) were eliminated due to being poorly measured in the vicinity of the beam stop. The H atom of the NH group of the title compound was located in a difference map and refined with the distance restraint N—H = 0.86 (2) Å; its isotropic displacement parameter was set to be 1.2*U*_eq_(N). The aromatic, methine, methylene and methyl H atoms were positioned geometrically with C—H = 0.93 - 0.98 Å, and allowed to ride on their parent atoms, with *U*_iso_(H) = 1.2*U*_eq_(C) for aromatic, methylene and methine, and 1.5*U*_eq_(C) for methyl.

### Crystal data

**Table:**

C_13_H_19_NO_3_S	*F*(000) = 1152
*M**_r_* = 269.36	*D*_x_ = 1.279 Mg m^−^^3^
Orthorhombic, *A**b**a*2	Mo *K*α radiation, λ = 0.71073 Å
Hall symbol: A 2 -2ac	Cell parameters from 3418 reflections
*a* = 17.2644 (12) Å	θ = 2.3–24.7°
*b* = 20.4707 (16) Å	µ = 0.23 mm^−^^1^
*c* = 7.9139 (5) Å	*T* = 296 K
*V* = 2796.9 (3) Å^3^	Prism, light brown
*Z* = 8	0.29 × 0.12 × 0.09 mm

### Data collection

**Table:**

Bruker APEXII CCD diffractometer	1945 reflections with *I* > 2σ(*I*)
Radiation source: sealed tube	*R*_int_ = 0.047
graphite	θ_max_ = 28.3°, θ_min_ = 3.0°
φ and ω scans	*h* = −23→23
10601 measured reflections	*k* = −23→27
2704 independent reflections	*l* = −10→6

### Refinement

**Table:**

Refinement on *F*^2^	Secondary atom site location: difference Fourier map
Least-squares matrix: full	Hydrogen site location: inferred from neighbouring sites
*R*[*F*^2^ > 2σ(*F*^2^)] = 0.048	H atoms treated by a mixture of independent and constrained refinement
*wR*(*F*^2^) = 0.136	*w* = 1/[σ^2^(*F*_o_^2^) + (0.0633*P*)^2^ + 1.387*P*] where *P* = (*F*_o_^2^ + 2*F*_c_^2^)/3
*S* = 1.02	(Δ/σ)_max_ < 0.001
2704 reflections	Δρ_max_ = 0.17 e Å^−^^3^
167 parameters	Δρ_min_ = −0.27 e Å^−^^3^
2 restraints	Absolute structure: Flack (1983), 829 Freidel pairs
Primary atom site location: structure-invariant direct methods	Flack parameter: −0.05 (12)

### Special details

**Table:**

Geometry. Bond distances, angles *etc*. have been calculated using the rounded fractional coordinates. All su's are estimated from the variances of the (full) variance-covariance matrix. The cell e.s.d.'s are taken into account in the estimation of distances, angles and torsion angles
Refinement. Refinement on *F*^2^ for ALL reflections except those flagged by the user for potential systematic errors. Weighted *R*-factors *wR* and all goodnesses of fit *S* are based on *F*^2^, conventional *R*-factors *R* are based on *F*, with *F* set to zero for negative *F*^2^. The observed criterion of *F*^2^ > σ(*F*^2^) is used only for calculating -*R*-factor-obs *etc*. and is not relevant to the choice of reflections for refinement. *R*-factors based on *F*^2^ are statistically about twice as large as those based on *F*, and *R*-factors based on ALL data will be even larger.

### Fractional atomic coordinates and isotropic or equivalent isotropic displacement parameters (Å^2^)

**Table:**

	*x*	*y*	*z*	*U*_iso_*/*U*_eq_	
S1	0.16185 (4)	0.09694 (5)	0.20157 (12)	0.0614 (3)	
O1	0.09659 (16)	0.11080 (14)	0.3056 (4)	0.0832 (11)	
O2	0.23822 (17)	0.11611 (14)	0.2537 (4)	0.0901 (11)	
O3	0.16806 (14)	−0.18793 (14)	0.1087 (4)	0.0822 (11)	
N1	0.14869 (13)	0.13120 (14)	0.0241 (4)	0.0556 (10)	
C1	0.0682 (2)	0.20104 (16)	−0.1482 (5)	0.0697 (15)	
C2	−0.0103 (3)	0.21102 (18)	−0.2295 (6)	0.0850 (16)	
C3	−0.0294 (2)	0.15634 (19)	−0.3505 (5)	0.0710 (16)	
C4	−0.0226 (2)	0.09157 (18)	−0.2642 (6)	0.0783 (16)	
C5	0.0557 (2)	0.08162 (16)	−0.1777 (6)	0.0724 (13)	
C6	0.07253 (15)	0.13658 (15)	−0.0581 (4)	0.0501 (10)	
C7	0.16495 (15)	0.01229 (17)	0.1741 (4)	0.0515 (10)	
C8	0.10900 (15)	−0.0285 (2)	0.2421 (4)	0.0613 (13)	
C9	0.11159 (16)	−0.09474 (19)	0.2187 (5)	0.0621 (11)	
C10	0.17072 (17)	−0.12166 (19)	0.1247 (5)	0.0576 (11)	
C11	0.22723 (18)	−0.08270 (18)	0.0575 (6)	0.0693 (16)	
C12	0.22479 (17)	−0.01640 (19)	0.0830 (5)	0.0694 (15)	
C13	0.2233 (3)	−0.2198 (2)	0.0041 (8)	0.111 (2)	
HN1	0.1894 (15)	0.1299 (18)	−0.037 (4)	0.0740*	
H1A	0.10810	0.20280	−0.23440	0.0840*	
H1B	0.07780	0.23610	−0.06840	0.0840*	
H2A	−0.04970	0.21310	−0.14230	0.1020*	
H2B	−0.01050	0.25220	−0.29000	0.1020*	
H3A	−0.08170	0.16190	−0.39270	0.0860*	
H3B	0.00570	0.15780	−0.44610	0.0860*	
H4A	−0.03020	0.05720	−0.34680	0.0940*	
H4B	−0.06340	0.08780	−0.18040	0.0940*	
H5A	0.05530	0.04060	−0.11610	0.0870*	
H5B	0.09620	0.07930	−0.26240	0.0870*	
H6	0.03280	0.13630	0.03040	0.0600*	
H8	0.06880	−0.01050	0.30500	0.0740*	
H9	0.07370	−0.12140	0.26590	0.0750*	
H11	0.26720	−0.10110	−0.00540	0.0830*	
H12	0.26380	0.00980	0.03860	0.0830*	
H13A	0.22010	−0.20250	−0.10840	0.1670*	
H13B	0.21260	−0.26580	0.00190	0.1670*	
H13C	0.27430	−0.21260	0.04830	0.1670*	

### Atomic displacement parameters (Å^2^)

**Table:**

	*U*^11^	*U*^22^	*U*^33^	*U*^12^	*U*^13^	*U*^23^
S1	0.0628 (4)	0.0785 (6)	0.0429 (4)	0.0052 (4)	−0.0071 (4)	−0.0091 (5)
O1	0.0961 (19)	0.106 (2)	0.0476 (16)	0.0250 (15)	0.0154 (14)	−0.0076 (15)
O2	0.0793 (17)	0.107 (2)	0.084 (2)	−0.0051 (15)	−0.0333 (15)	−0.0189 (17)
O3	0.0877 (18)	0.0690 (18)	0.090 (2)	−0.0011 (13)	0.0110 (15)	0.0140 (15)
N1	0.0457 (13)	0.0707 (19)	0.0503 (17)	−0.0032 (12)	0.0027 (11)	−0.0033 (14)
C1	0.096 (3)	0.0442 (18)	0.069 (3)	−0.0004 (17)	−0.0043 (19)	−0.0053 (19)
C2	0.110 (3)	0.066 (2)	0.079 (3)	0.024 (2)	−0.020 (2)	0.002 (2)
C3	0.082 (2)	0.074 (3)	0.057 (3)	0.0093 (19)	−0.0133 (18)	0.0056 (19)
C4	0.082 (2)	0.064 (2)	0.089 (4)	−0.0068 (18)	−0.033 (2)	0.000 (2)
C5	0.086 (2)	0.0413 (18)	0.090 (3)	0.0029 (17)	−0.028 (2)	−0.0018 (19)
C6	0.0466 (14)	0.057 (2)	0.0467 (18)	0.0002 (12)	0.0011 (13)	0.0052 (14)
C7	0.0403 (13)	0.075 (2)	0.0391 (19)	0.0071 (13)	−0.0031 (12)	0.0041 (15)
C8	0.0380 (14)	0.098 (3)	0.048 (2)	0.0078 (15)	0.0035 (13)	0.0058 (18)
C9	0.0442 (14)	0.084 (2)	0.058 (2)	−0.0074 (15)	0.0043 (16)	0.016 (2)
C10	0.0542 (18)	0.070 (2)	0.0487 (19)	0.0028 (16)	−0.0023 (15)	0.0097 (17)
C11	0.064 (2)	0.074 (3)	0.070 (3)	0.0076 (17)	0.0265 (19)	0.007 (2)
C12	0.0581 (19)	0.082 (3)	0.068 (3)	−0.0029 (16)	0.0222 (17)	0.009 (2)
C13	0.122 (4)	0.091 (3)	0.121 (5)	0.019 (3)	0.035 (3)	−0.009 (3)

### Geometric parameters (Å, °)

**Table:**

S1—O1	1.424 (3)	C11—C12	1.373 (5)
S1—O2	1.436 (3)	C1—H1A	0.9700
S1—N1	1.586 (3)	C1—H1B	0.9700
S1—C7	1.747 (4)	C2—H2A	0.9700
O3—C10	1.363 (5)	C2—H2B	0.9700
O3—C13	1.421 (6)	C3—H3A	0.9700
N1—C6	1.471 (4)	C3—H3B	0.9700
N1—HN1	0.85 (3)	C4—H4A	0.9700
C1—C2	1.514 (6)	C4—H4B	0.9700
C1—C6	1.502 (5)	C5—H5A	0.9700
C2—C3	1.510 (6)	C5—H5B	0.9700
C3—C4	1.496 (6)	C6—H6	0.9800
C4—C5	1.529 (5)	C8—H8	0.9300
C5—C6	1.499 (5)	C9—H9	0.9300
C7—C12	1.390 (4)	C11—H11	0.9300
C7—C8	1.386 (4)	C12—H12	0.9300
C8—C9	1.369 (6)	C13—H13A	0.9600
C9—C10	1.378 (5)	C13—H13B	0.9600
C10—C11	1.368 (5)	C13—H13C	0.9600
			
O1—S1—O2	120.39 (18)	C3—C2—H2A	109.00
O1—S1—N1	108.09 (16)	C3—C2—H2B	109.00
O1—S1—C7	107.08 (16)	H2A—C2—H2B	108.00
O2—S1—N1	105.37 (16)	C2—C3—H3A	110.00
O2—S1—C7	106.18 (15)	C2—C3—H3B	110.00
N1—S1—C7	109.43 (16)	C4—C3—H3A	110.00
C10—O3—C13	119.2 (3)	C4—C3—H3B	110.00
S1—N1—C6	123.6 (2)	H3A—C3—H3B	108.00
S1—N1—HN1	112 (2)	C3—C4—H4A	109.00
C6—N1—HN1	119 (2)	C3—C4—H4B	109.00
C2—C1—C6	111.4 (3)	C5—C4—H4A	109.00
C1—C2—C3	111.4 (3)	C5—C4—H4B	109.00
C2—C3—C4	110.5 (3)	H4A—C4—H4B	108.00
C3—C4—C5	113.1 (3)	C4—C5—H5A	109.00
C4—C5—C6	110.7 (3)	C4—C5—H5B	110.00
N1—C6—C5	113.4 (3)	C6—C5—H5A	109.00
C1—C6—C5	110.5 (3)	C6—C5—H5B	109.00
N1—C6—C1	108.7 (2)	H5A—C5—H5B	108.00
C8—C7—C12	117.7 (3)	N1—C6—H6	108.00
S1—C7—C8	121.9 (2)	C1—C6—H6	108.00
S1—C7—C12	120.4 (3)	C5—C6—H6	108.00
C7—C8—C9	121.4 (3)	C7—C8—H8	119.00
C8—C9—C10	119.6 (3)	C9—C8—H8	119.00
O3—C10—C11	124.6 (3)	C8—C9—H9	120.00
O3—C10—C9	115.0 (3)	C10—C9—H9	120.00
C9—C10—C11	120.3 (4)	C10—C11—H11	120.00
C10—C11—C12	119.8 (3)	C12—C11—H11	120.00
C7—C12—C11	121.1 (3)	C7—C12—H12	119.00
C2—C1—H1A	109.00	C11—C12—H12	120.00
C2—C1—H1B	109.00	O3—C13—H13A	109.00
C6—C1—H1A	109.00	O3—C13—H13B	109.00
C6—C1—H1B	109.00	O3—C13—H13C	109.00
H1A—C1—H1B	108.00	H13A—C13—H13B	110.00
C1—C2—H2A	109.00	H13A—C13—H13C	110.00
C1—C2—H2B	109.00	H13B—C13—H13C	110.00
			
O1—S1—N1—C6	−36.4 (3)	C1—C2—C3—C4	54.1 (4)
O2—S1—N1—C6	−166.3 (3)	C2—C3—C4—C5	−53.2 (5)
C7—S1—N1—C6	79.9 (3)	C3—C4—C5—C6	54.4 (5)
N1—S1—C7—C12	65.7 (3)	C4—C5—C6—N1	−177.6 (3)
O2—S1—C7—C8	132.0 (3)	C4—C5—C6—C1	−55.4 (4)
N1—S1—C7—C8	−114.7 (3)	S1—C7—C12—C11	−178.7 (3)
O1—S1—C7—C8	2.2 (3)	C8—C7—C12—C11	1.7 (5)
O2—S1—C7—C12	−47.6 (3)	S1—C7—C8—C9	179.5 (3)
O1—S1—C7—C12	−177.4 (3)	C12—C7—C8—C9	−1.0 (5)
C13—O3—C10—C11	−5.6 (6)	C7—C8—C9—C10	−0.4 (5)
C13—O3—C10—C9	175.5 (4)	C8—C9—C10—C11	1.1 (6)
S1—N1—C6—C1	144.0 (3)	C8—C9—C10—O3	−179.9 (3)
S1—N1—C6—C5	−92.7 (3)	O3—C10—C11—C12	−179.2 (4)
C2—C1—C6—N1	−177.5 (3)	C9—C10—C11—C12	−0.4 (6)
C6—C1—C2—C3	−57.0 (4)	C10—C11—C12—C7	−1.1 (6)
C2—C1—C6—C5	57.5 (4)		

### Hydrogen-bond geometry (Å, °)

**Table:**

*D*—H···*A*	*D*—H	H···*A*	*D*···*A*	*D*—H···*A*
N1—HN1···O2^i^	0.85 (3)	2.09 (3)	2.913 (4)	161 (3)

Symmetry codes: (i) −*x*+1/2, *y*, *z*−1/2.
